# Elevation of ω-3 Polyunsaturated Fatty Acids Attenuates PTEN-deficiency Induced Endometrial Cancer Development through Regulation of COX-2 and PGE_2_ Production

**DOI:** 10.1038/srep14958

**Published:** 2015-10-15

**Authors:** Jinshun Pan, Lixian Cheng, Xinyun Bi, Xin Zhang, Shanshan Liu, Xiaoming Bai, Fanghong Li, Allan Z. Zhao

**Affiliations:** 1 The Center of Metabolic Disease Research, Nanjing Medical University, Nanjing, Jiangsu Province 210029, China; 2 Cancer Center, Department of Pathology, Nanjing Medical University, Nanjing, Jiangsu Province 210029, China

## Abstract

Endometrial cancer is one of the most common gynecologic malignancies. Phosphatase and tensin homologue (PTEN)-mutation is frequently identified in endometrial cancer patients. Although high dietary intake of ω-3 polyunsaturated fatty acids (PUFAs) has been associated with reduced risk of endometrial cancer, the underlying mechanisms is still unknown. To this end, we evaluated the impact of ω-3 PUFAs using several endometrial cancer cellular and animal models. While ~27% and 40% of heterozygotic PTEN mutant mice developed endometrial cancer and atypical complex hyperplasia, respectively, none of the PTEN^+/−^ mice developed cancer when we overexpressed an mfat-1 transgene, which allowed endogenous production of ω-3 PUFAs. Fish oil-enriched diet or expression of mfat-1 transgene significantly inhibited the growth of xenograft tumor derived from RL95-2 cells bearing a PTEN null mutation. At cellular level, ω-3 PUFAs treatment decreased the viability of RL95-2 cells, *AKT* phosphorylation, and cyclin D1 expression. These molecular events are primarily mediated through reduction of cyclooxygenase-2 (COX-2) expression and prostaglandin E_2_ (PGE_2_) production. Exogenous PGE_2_ treatment completely blunted the impact of ω-3 PUFAs on endometrial cancer. Thus, we revealed the direct inhibitory effects of ω-3 PUFAs on endometrial cancer development and the underlying mechanisms involving reduction of COX-2 and PGE_2_.

Endometrial cancer is the most common gynecologic malignancy and the fourth most common cancer for women worldwide. There are nearly 200,000 cases diagnosed each year, comprising 6% of female cancers^1^,^2^,^3^. Mutations of the tumor suppressor gene phosphatase and tensin homologue (PTEN) were found to play a significant role in the pathogenesis of endometrial cancer, with PTEN mutation present in approximately 40–80% of cases^3^,^4^,^5^.

The PTEN gene is located on chromosome 10q23, a genomic region that suffers loss of heterozygosity in many human cancers. Somatic deletions or mutations of this gene have been identified in many human sporadic cancers, such as endometrial cancer, colorectal cancer, and glioblastoma^4^,^6^,^7^. In particular, loss of PTEN function by mutational mechanism has been investigated as an early event in endometrial tumorigenesis^5^. Consistent with the clinical observations, haploid deficiency of PTEN have a high incidence of endometrial neoplasia in mice^8^,^9^. Thus, targeting PTEN-deficiency initiated may represent a new therapeutic strategy for the prevention and treatment of this malignant disease.

ω-3 and ω-6 polyunsaturated fatty acids (PUFAs) are essential fatty acids necessary for human health, all of which have to be obtained through diets due to the inability of mammals synthesizing these fatty acids^10^. Epidemiological literatures on the linkage between ω-3 PUFAs and cancer incidence, including cross-sectional and migrational studies, have revealed a protective effect of ω-3 PUFAs and a promoting effect of ω-6 PUFAs on the development of cancers^11^,^12^. Specifically, typical modern western diets are high in ω-6 but low in ω-3 PUFAs, and are positively associated with tumorigenesis and poor prognosis of cancers^11^,^13^. Dietary intake of high levels of long chain ω-3 PUFAs has been shown to reduce various cancers and alleviate their complications^14^,^15^. Clinically, long-term high intake of diets or supplements enriched in eicosapentaenoic acid (EPA) and docosahexaenoic acid (DHA) were associated with lower risk of endometrial cancer^16^,^17^. Dietary ω-3 PUFAs significantly attenuated endometrial cancer cell growth in xenograft models^18^. Therefore, high circulating and tissue contents of ω-3 PUFAs may be an important tool in the prevention and treatment of cancer pathogenesis^11^,^19^.

We previously reported a transgenic mouse model overexpressing a *C. elegans* gene, mfat-1, encoding an ω-3 fatty acid desaturase^20^. This enzyme can produce ω-3 PUFAs endogenously by converting ω-6 to ω-3 PUFAs, which enables the investigation of the biological properties of ω-3 PUFAs without the need of lengthy feeding of fish oil. Furthermore, this model also makes it possible to use genetic approach by, for example, crossing the mfat-1 transgenics with the haploid PTEN-deficient mice. Such genetic approach also complements very well the xenogenic model with endometrial cancer RL95-2 cells, a PTEN-deficient cell line. With these animal models, we can interrogate the impact and underlying mechanisms of ω-3 PUFAs on spontaneously developed PTEN-deficiency-induced primary lesions. The potential positive outcomes of our studies may benefit the patients with PTEN-deficient endometrial cancer.

## Results

### ω-3 PUFAs attenuates PTEN-deficiency induced primary endometrial cancer development

**Figure 1:**
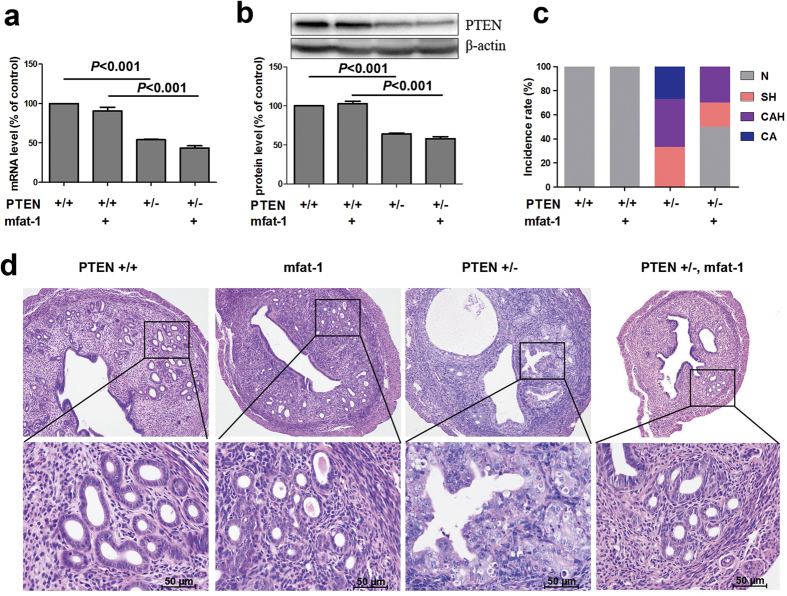
Endogenously produced ω-3 PUFAs attenuates PTEN-deficiency induced primary endometrial cancer development. (**a**) The uteri of mice were used for identification of PTEN deletion. Real-time PCR analysis showing mRNA level in uterus of PTEN^+/−^ mice was the half of the PTEN^+/+^ mice. (**b**) Western blotting analysis found the decreased level of PTEN protein expression in uterus of PTEN^+/−^ mice. β-actin was used as internal control. Data are means ± SD. n = 6. (**c**). Hematoxylin-eosin (HE) staining was used to evaluate histopathological grades of endometrial neoplasia. Incidence of endometrial hyperplasia and cancer in four groups were shown. (PTEN^+/+^, n = 13; mfat-1, n = 11; PTEN^+/−^, n = 15; mfat-1;PTEN^+/−^, n = 10) (**d**). HE staining of uterus sections showing histopathological grades of endometrial neoplasia in mice, according to the criteria described in text. The strains of PTEN^+/+^ and mfat-1 were served as littermate control. Scale bars = 50 μm.

To investigate the impact of ω-3 PUFAs on primary endometrial cancer development, we genetically crossed the mfat-1 transgenic mice with PTEN^+/−^ mice to allow this enzyme to produce ω-3 PUFAs in the tissues^20^,^21^. The levels of PTEN mRNA and protein in the uterus of PTEN^+/−^ mice were about half of the PTEN^+/+^ mice (Fig. 1a,b), confirming the haploid deficiency of PTEN expression. Analysis of fatty acid compositions confirms the activity of mfat-1 protein, with a significant decrease in arachidonic acid (AA), a concomitant increase in EPA and DHA levels, and a significantly decreased ratio of ω-6/ω-3 PUFAs compared with the mice lacking mfat-1 expression (PTEN^+/+^, PTEN^+/−^) (Table 1).

Consistent with previous reports^8^,^9^, high incidence of endometrial complex atypical hyperplasia (CAH, 40%) and carcinoma (CA, ~27%) were observed in the uterus of PTEN^+/−^ mice (Fig. 1c,d). In contrast, analysis of serial sections of uterus samples did not reveal any malignant lesion in any of the mfat-1;PTEN^+/−^ animals and the CAH incidence was reduced to 30% within this group (Fig. 1c,d). Interestingly, even the rate of simple hyperplasia (SH) markedly dropped from ~33% to 20%. In the control groups (wild-type mice or mfat-1 transgenics), the uterus tissues were completely normal with no identifiable hyperplasia (Fig. 1c,d). These results suggest that elevated tissue levels of ω-3 PUFAs suppress PTEN-deficiency induced endometrial cancer development.

### ω-3 PUFAs inhibit endometrial cancer cell growth in xenograft models

**Figure 2:**
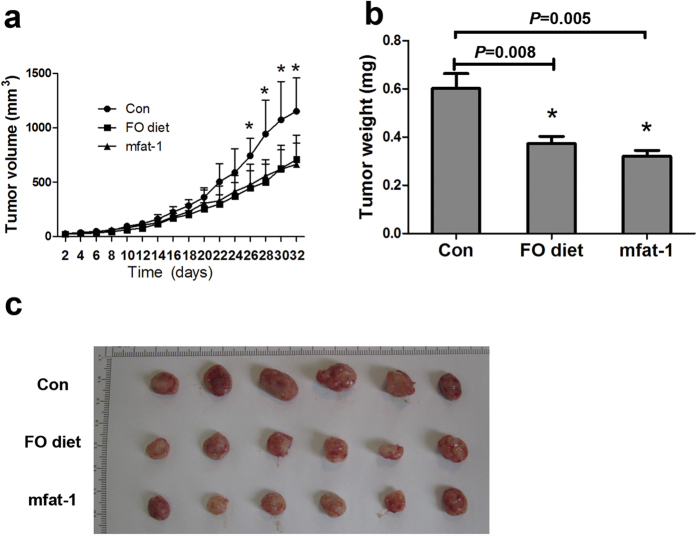
Exogenous and endogenously produced ω-3 PUFAs inhibit endometrial cancer cell growth in xenograft models. Four-week-old female nude mice were randomized into three groups (n = 10). Group 1 was implanted with RL95-2-GFP cells and fed with normal diet. Group 2 was implanted with RL95-2-GFP cells and fed with high ω-3 PUFAs diet (normal diet mixed with 5% FO). Group 3 was implanted with RL95-2-mfat-1 cells and fed with normal diet. The tumor growth curve (**a**) and a comparison of average tumor weight (**b**) on the final day among three groups are shown. Data are means ± SD. n = 10. ^*^P < 0.05 compared with respective controls. (**c**) Representative images of tumors from each group.

As a further step of demonstrating the beneficial effect of ω-3 PUFAs against endometrial cancer cell development *in vivo*, we studied this issue in a xenograft model. We infected RL95-2 cells, a human endometrial carcinoma line containing PTEN null mutation, with lentivirus carrying the mfat-1 gene (termed “RL95-2-mfat-1” cells). As a control, we also generated a green fluorescent protein (GFP) expressing line, RL95-2-GFP. The expression of mfat-1 protein endogenously converted ω-6 PUFAs to ω-3 PUFAs in the RL95-2 cells (Table 1). Following the establishment of these cell lines, we established endometrial tumor xenografts by subcutaneously implanting these RL95-2 cell lines into immune-deficient nude mice. Although the mice implanted with the RL95-2-mfat-1 cells were maintained on regular diet, the mice implanted with the control RL95-2-GFP cells were fed either regular diet or regular diet mixed with 5% fish-oil (FO). Analysis of fatty acid composition identified significantly decreased ω-6 and elevated ω-3 PUFAs content in the tumors derived from the mice either fed 5% FO-containing diet or implanted with RL95-2-mfat-1 cells (Table 1). Analysis of implanted human tumor xenografts revealed that elevated tissue contents of ω-3 PUFAs significantly reduced tumor growth as measured by tumor volume and weight (Fig. 2), regardless if the source of ω-3 PUFAs was coming from the mfat-1 expression or dietary intake.

### ω-3 PUFAs inhibit *Akt* phosphorylation and cyclin D1 expression in primary endometrial cancer

**Figure 3:**
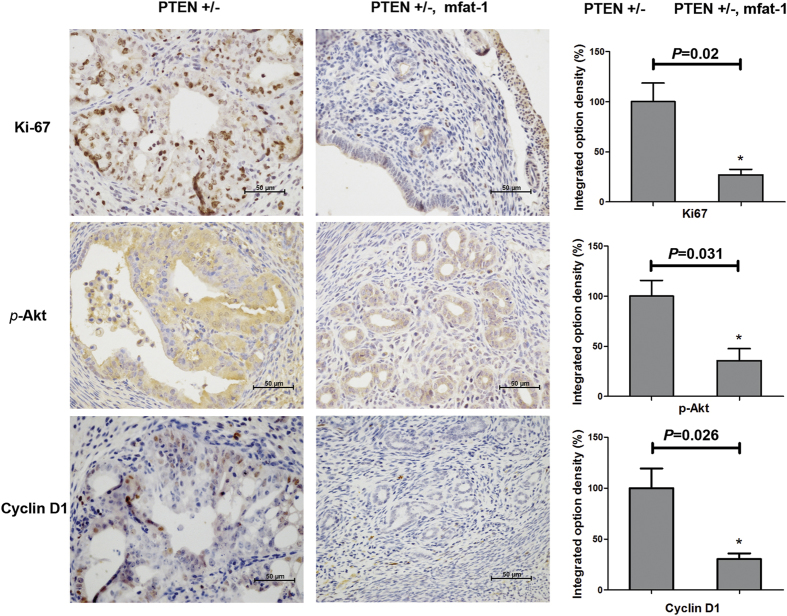
Endogenously produced ω-3 PUFAs inhibit Ki-67, p-*Akt* and cyclin D1 *in vivo.* Immunohistochemistry studies showed the decreased level of Ki-67, p-*Akt*, and cyclin D1 in malignant tissue in mfat-1;PTEN^+/−^ mice, compared with Pten^+/−^ mice. Scale bars = 50 μm. Immunostained expression levels of proteins were quantitated using Image Pro Plus 6.0 software (Media Cybernetics Inc., New York, USA).

The constitutive activation of phosphoinositide-3 kinase (PI3K)-*Akt* pathway is a direct consequence of PTEN null mutation, and the primary cause of malignant cell proliferation. Through immunohistochemistry analysis, we found that PTEN deficiency-induced *Akt* phosphorylation and cyclin D1 expression were significantly suppressed in endometrial tissues of PTEN^+/−^ mice when mfat-1 was transgenically expressed (Fig. 3). Meanwhile, the number of Ki-67 cells, a molecular marker of cell proliferation, was also sharply reduced in the mfat-1;PTEN^+/−^ mice (Fig. 3). Thus, elevation of ω-3 PUFAs in the uterine tissues strongly decreased PTEN-deficiency induced *Akt* phosphorylation and cyclin D1 expression in malignant endometrial cancer tissues.

### ω-3 PUFAs inhibit endometrial cancer cell growth *in vitro*

**Figure 4:**
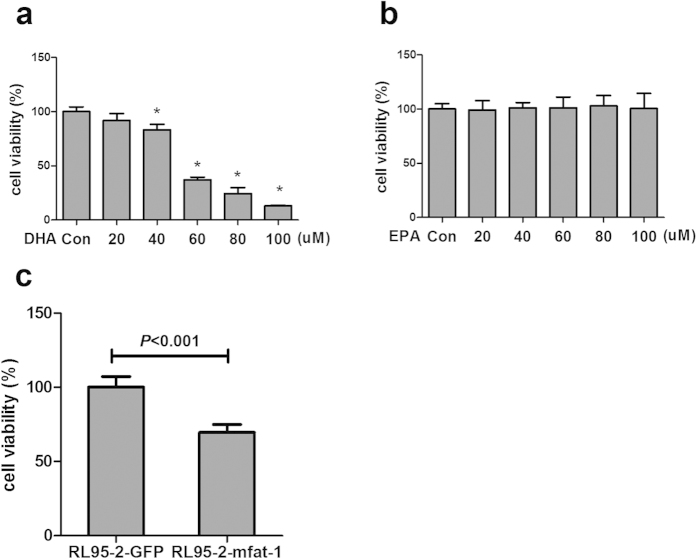
Exogenous and endogenously produced ω-3 PUFAs inhibit endometrial cancer cell growth *in vitro.* (**a**,**b**) RL95-2 cells grown in 96-well plates were treated with various concentrations of DHA (**a**) or EPA (**b**) Data were analyzed by one-way ANOVA followed by Dunnett’s test, and the linear trend was significant (*P *< 0.01). (**c**) The RL95-2-GFP and RL95-2-mfat-1 cells were subjected to the cell growth assay. The mfat-1 expressed cell viability was significantly inhibited. Data are means ± SD. n = 6.

To investigate if the effect of ω-3 PUFAs is directly acted on endometrial cancer cells, we initially examined the impact of adding EPA or DHA to the cultured RL95-2 cells. DHA inhibited RL95-2 cell viability in a dose-dependent manner (Fig. 4a). At the highest concentration (100 μM) tested, DHA caused a 5-fold reduction of cell viability in RL95-2 cells (Fig. 4a). Interestingly, addition of EPA did not significantly change the growth curve of the cultured cells (Fig. 4b). Thus, the primary effector of the ω-3 PUFAs mixture appears to be DHA in inhibiting RL95-2cell growth. Consistent with this observation, we also found mfat-1 expression significantly suppressed cell viability (Fig. 4c). Flow cytometry analysis of cell cycle further revealed that mfat-1 expression caused a G1 arrest in cell cycle in RL95-2 cell line (Table 2). Taken together, these results indicate that ω-3 PUFA, particularly DHA, and mfat-1 expression can directly inhibit endometrial cancer cell growth.

### Metabolomic analysis of eicosanoids in RL95-2 cells

The differential effects of ω-6 and ω-3 PUFAs on cancers may be mediated by their eicosanoid metabolites. As the initial step of addressing this issue, we analyzed some of the eicosanoid metabolites induced by ω-6 or ω-3 PUFA treatment. Using an LC−MS/MS-based method, we analyzed 31 AA metabolites and 15 DHA-derived products (Table 3). As one might expect, 29 of the 31 identified AA-derived metabolites were increased in RL95-2 cells following 50 μM AA treatment, chief among which prostaglandin E_2_ (PGE_2_), LTB_4_, 5-HETE, 12-HETE, 15-HETE, 20-HETE, 11,12-EET, and 14,15-EET, which are known to promote cancer growth^22^,^23^,^24^,^25^,^26^,^27^,^28^, were sharply elevated compared with the control group. Interestingly, the expression of mfat-1 and DHA treatment, though both elevate DHA in RL95-2 cells, led to differential production of eicosanoids. For example, mfat-1 expression decreased only eight of the AA-derived eicosanoids compared with the RL95-2-GFP group, whereas incubation of DHA caused reduction of PGE_2_. In addition, thirteen of DHA-derived eicosanoids were elevated following DHA treatment. On the other hand, expression of mfat-1 transgene only increased the production of 13-HDoHE and 19,20-EDP. Despite these differences, both treatments led to reduction of AA-derived PGE_2_. Accordingly, DHA treatment and mfat-1 expression significantly decreased PGE_2_ production by 3.6 fold and 3.2 fold, respectively (Table 3).

### cyclooxygenase-2 (COX-2) expression and PGE_2_ production were involved in ω-3 PUFAs-mediated attenuation in PTEN-deficient endometrial cancer

**Figure 5:**
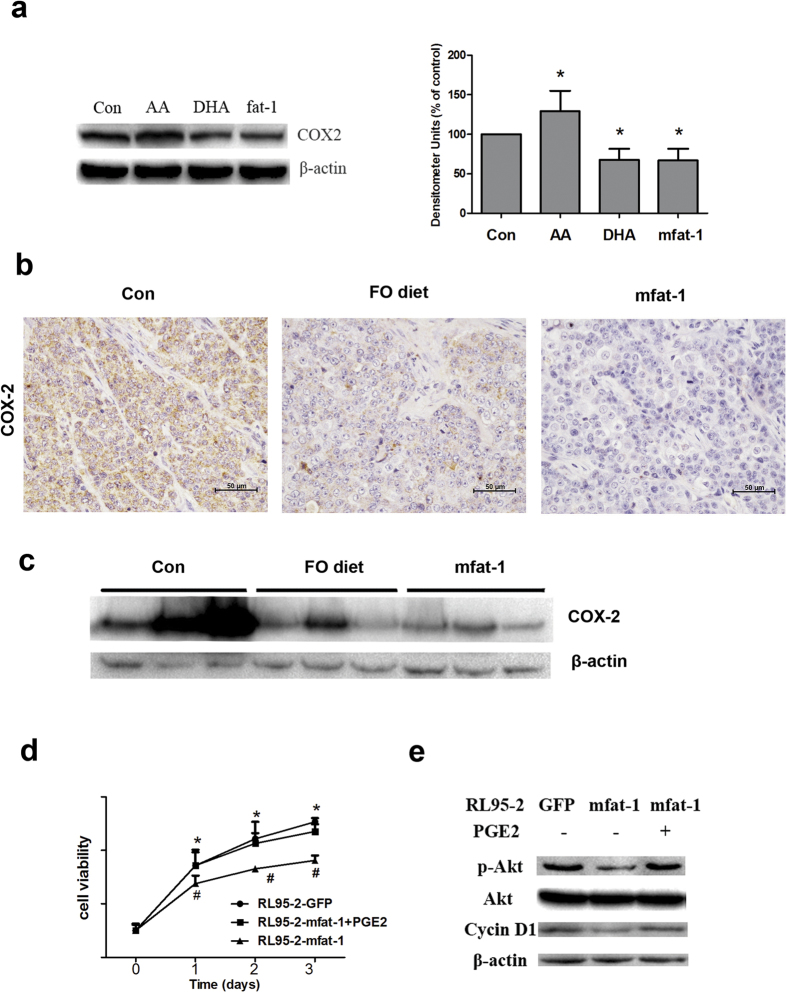
Exogenous and endogenously produced ω-3 PUFAs inhibit COX-2 expression and PGE_2_ production. (**a**) Detection of COX-2 by western blotting. RL95-2-GFP and RL95-2-mfat-1 cells were serum-starved for 12 h, followed by treatment with the 50 μM AA or 60 μM DHA for 24 h. Data are means ± SD. n = 3. (**b**) In nude mice, immunohistochemical staining showed both 5% FO diet and mfat-1 expression inhibited COX-2 expression in implanted tumors. (**c**) Levels of COX-2 in implanted tumors were determined by western blotting. (**d**) Growth of RL95-2-GFP and RL95-2-mfat-1 cells. The viability of mfat-1 expressed cell was significantly inhibited. ^*^P < 0.05 compared with RL95-2-GFP cells. PGE_2_ treatment partially counteracted mfat-1-induced proliferation arrest and showed no significant difference with RL95-2-GFP cells. ^#^*P *< 0.05 compared with RL95-2-mfat-1 cells. Data are means ± SD. n = 6. (**e**) Levels of p-*Akt* and cyclin D1 were detect**e**d by western blotting. Total *Akt* and β-actin were used as respective controls.

As a bioactive lipid that elicits a wide range of biological effects associated with inflammation and cancer^29^, PGE_2_ is derived from AA as a result of the sequential enzymatic reactions of COX-2 and PGE synthase. To interrogate how ω-3 PUFAs suppress cell proliferation through eicosanoid metabolites, we evaluated the expression of COX-2 following the treatment with AA or DHA. Although incubation of RL95-2 cells with AA significantly elevated COX-2 expression, DHA treatment significantly decreased COX-2 expression (Fig. 5a). The expression of mfat-1 mimicked the effect of DHA on COX-2 regulation (Fig. 5a). In the xenografts, both feeding of 5% FO-containing diet and mfat-1 expression inhibited COX-2 expression (Fig. 5b,c).

Addition of PGE_2_ to RL95-2 cells had a profound effect on ω-3 PUFAs-suppressed cell proliferation, *Akt* phosphorylation, and cyclin D1 expression. Cell proliferation assays showed that mfat-1 expression decreased cell proliferation, compared with RL95-2-GFP cells. Consistent with this observation, while exogenous PGE_2_ treatment partially counteracted mfat-1-induced proliferation arrest (Fig. 5d). As shown in Fig. 5e, *Akt* phosphorylation and cyclin D1 expression were significantly decreased in RL95-2-mfat-1 cells compared with RL95-2-GFP cells. However, addition of PGE_2_ counteracted the impact of ω-3 PUFAs on *Akt* phosphorylation and cyclin D1 expression, suggesting that regulation of COX-2 expression and PGE_2_ production played a major role in ω-3 PUFAs-mediated growth inhibition of endometrial cancer.

## Discussion

The overall aim of this study is to investigate the impact and the underlying mechanisms of ω-3 PUFAs on endometrial cancer, particularly the type carrying genetic mutation in PTEN, whose primary function is to oppose PI3K activity^7^,^30^,^31^. PTEN-deficiency is the most common genetic mutation detected thus far in endometrial cancers^3^,^4^,^5^. Mice carrying heterozygotic mutation of PTEN also have a high incidence of endometrial neoplasia^8^,^9^. We took advantage of a previously described transgenic model, mfat-1, that allows production of ω-3 PUFAs in all tissues including the uterus of PTEN haploid deficient mice. In contrast to the high incidence of endometrial neoplasm (~27%) and atypical hyperplasia (40%) associated with PTEN heterozygotic deficiency^8^,^9^,^21^,^32^, the expression of mfat-1 in the PTEN^+/−^ mice displayed no malignant endometrial lesion and significantly reduced incidence of atypical complex hyperplasia (30%). The constitutive activation of PI3K-Akt pathway and the resulting increased expression of cyclin D1 is a critical mechanism of aggressive cell proliferation in the context of PTEN-deficiency^33^,^34^,^35^,^36^. Indeed, Akt1 deficiency is sufficient to inhibit the development of endometrial carcinoma in PTEN^+/−^ mice^8^. Consistent with the pathology findings, the levels of phospho-Akt and cyclin D1 expression were greatly suppressed by elevated levels of ω-3 PUFAs as a result of mfat-1 expression. Such results derived from the primary endometrial carcinoma were also confirmed at cellular levels or in a xenograft model in which dietary addition of ω-3 PUFAs or mfat-1 expression inhibited the growth of cultured or implanted RL95-2 cells carrying a PTEN null mutation. Combined together, these data clearly demonstrate that ω-3 PUFAs can strongly suppress carcinogenesis and progression of endometrial cancer by inhibiting activation of PI3K/Akt/cyclin D1 signal pathway in the context of PTEN deficiency.

Mounting evidence has linked dietary gain of ω-3 PUFAs to the prevention or attenuation of progression of several cancers, such as colon^37^, breast^37^,^38^, and prostate cancers^39^. A series of recent studies have also shown that mfat-1 expression, which produces ω-3 PUFAs endogenously, also inhibited the growth of colon^40^, prostate^41^, liver^42^, and breast cancer^43^. However, these strong phenotypic results, including the aforementioned phenotypes of ω-3 PUFAs on endometrial cancer have not conclusively revealed how ω-3 PUFAs achieved these anti-tumor effects. We focused on the roles of metabolites derived from ω-3 and ω-6 PUFAs. Eicosanoids are widely believed to play important roles in carcinogenesis and aggressiveness of many cancers^23^,^42^,^44^,^45^. Out of the 31 AA metabolites and 15 DHA-derived products evaluated in our assays, we found that AA treatment increased AA-derived metabolites levels including 14,15-EET, LTB_4_, 5-HETE, 12-HETE, 15-HETE, 20-HETE, 11,12-EET, and PGE_2_, all of which have been shown to promote the development of many types of cancers^22^,^23^,^24^,^25^,^26^,^27^,^28^. In contrast, treatment with ω-3 PUFAs significantly decreased PGE_2_ levels while elevating the production of DHA-derived metabolites levels including 13-HDoHE and 19, 20-EDP.

PGE_2_ is a direct product of oxidization by COX-1 or COX-2. Previous studies have revealed elevated expression of COX-2 in various inflammatory diseases and human cancers^25^,^29^,^46^,^47^,^48^,^49^, including association with poor prognosis in patients suffering endometrial cancer^50^. Importantly, selective inhibitors of COX-2 resulted in significant antitumor effects in endometrial cancer patients^51^,^52^. In our studies, elevation of ω-3 PUFAs caused a significant inhibition of COX-2 expression *in vitro* and *in vivo.* Treatment of RL95-2 cells with exogenous PGE_2_ counteracted all of the ω-3 PUFAs-induced effects, including proliferation arrest, reduction of Akt phosphorylation and cyclin D1 expression. These data suggest that reduction of COX-2 expression and AA-derived metabolite, PGE_2,_ are important mechanisms underlying ω-3 PUFA-induced interruption of PI3K/Akt signal pathway.

In summary, this study provides the definitive *in vivo* evidence that ω-3 PUFAs strongly inhibit tumorigenesis and progression of PTEN-deficiency induced endometrial cancer through a mechanism involving reduction of COX-2 expression and PGE_2_ production. Such mechanisms oppose the constitutive activation of Akt phosphorylation, cyclin D1 expression. The suppressive effect of ω-3 PUFAs in the development and progression of endometrial cancer may offer novel prevention and treatment strategy of this malignant disease in women.

## Methods

### Animals

All experimental protocols were approved by the Research Ethics Committee of the Nanjing Medical University. All experiments were conducted in compliance with the guidelines for the care and use of laboratory animals and approved by Institutional Animal Care and Use Committee of Nanjing Medical University. The normal diet was from Xietong Biotechnology Co.Ltd (Jiangsu, China). The fish oil was from ShangHai HOPE industry Co. Ltd (Shanghai, China). The high ω-3 PUFAs diet was made by the normal diet mixed with 5% Fish-Oil (FO). The animals were fed food and water *ad libitum*, housed at 22 °C and a 12-h light-dark cycle.

### Materials

All cell culture reagents were purchased from Gibco BRL Technology. AA, EPA, DHA, propidium iodide (PI), and PUFA analytical standards were obtained from Sigma-Aldrich (St. Louis, MO, USA). Water-soluble tetrazolium (WST) reagent was from Dojindo Laboratories (Kumamoto, Japan). The following were commercially obtained antibodies: PTEN antibody, Ki-67 antibody, Cyclin D1 antibody, anti-phospho-*Akt* antibody (Ser473), *Akt* antibody (Cell Signaling Technology Danvers, MA, USA); anti-β-actin antibody (Abcam, Cambridge, UK); anti- COX-2 antibody and PGE_2_ (Cayman Chemical Co, Ann Arbor, MI, USA)

### Generation of mice with mfat-1;PTEN^+/−^

The PTEN^loxP/loxP^ mice^21^ and zona pellucida 3 (Zp3)-Cre transgenic mice were obtained from the Model Animal Research Center of Nanjing University (Nanjing, China). The mfat-1 transgenic mice were described in our previous studies^20^. All above strains of mice have a C57BL/6J genetic background. The PTEN gene was deleted specifically from oocytes of primary follicles by crossing Zp3-Cre mice with PTEN^loxP/loxP^ mice as previously described^53^. The female offspring was crossed with male mfat-1 transgenic mice to generate four different genotypes for experiments (PTEN^+/+^, mfat-1, PTEN^+/−^, mfat-1;PTEN^+/−^). The PTEN^+/+^ and mfat-1 mice were served as littermate control.

The uterus sections from the female mice at 26 weeks of age were subjected to histopathological analyses. Normal (N): the endometrium is covered with columnar lining epithelium with widely separated tubular glands embedded in a cellular stroma; Simple hyperplasia (SH): increased number of simple tubular glands or the glands covered by crowded hyperplastic epithelium; Complex atypical hyperplasia (CAH): enlarged and irregularly branching endometrial glands; Carcinoma (CA): glands with multilayer cells and extensive cribriform structures or sheets of large pleomorphic epithelial cells.

### PCR Analysis of PTEN and mfat-1 Genotypes

Tail genomic DNA samples were isolated and amplified by PCR following by a previously described protocol^20^. Sense primer (5′-CTCCTCTACTCCATTCTTCCC-3′) and anti-sense primer (5′-ACTCCCACCAATGAACAAAC-3′) were used to detect the PTEN^loxp/loxp^ allele, sense primer (5′-GTCACCAGGATGCTTCTGAC-3′) and anti-sense primer (5′-TGGCAAGCTTTCAACTTGTTT-3′) were used to detect PTEN^+/−^, sense primer (5′-GGACCTGGTGAAGAGCATCCG-3′) and anti-sense primer (5′-GCCGTCGCAGAAGCCAAAC-3′) were used to detect the mfat-1, and sense primer (5′-CAGATGAGGTTTGAGGCCACAG-3′) and anti-sense primer (5′-CAGGTGTTATAAGCAATCCC-3′) were used to detect Zp3-Cre transgene. Amplified fragments of 325 bp, 750 bp, 438 bp, and ~500 bp were obtained, respectively.

### Tumor xenograft models

Four-week-old female *BALB/c* nude mice were injected with 5 × 10^6^/0.1 ml of RL95-2 cells into the flanks. The mice were randomized into three groups (n = 10). Group 1 was implanted with RL95-2-GFP cells and fed with normal diet. Group 2 was implanted with RL95-2-GFP cells and fed with high ω-3 PUFAs diet (normal diet mixed with 5% FO). Group 3 was implanted with RL95-2-mfat-1 cells and fed with normal diet. Bi-dimensional tumor measurements were taken every two days. Tumor volume was measured along two major axes using calipers. Tumor volume (mm^3^) was calculated as follows: V = 1/2 LW^2^ (L: length, W: width).

### Cell culture and proliferation assays

The endometrial cancer cell lines RL95-2 was obtained from American Type Culture Collection (ATCC). RL95-2 cells were cultured in Dulbecco’s modified Eagle’s medium (DMEM) with 5% fetal calf serum, 100 IU/ml penicillin and 100 μg/ml streptomycin at 37 °C with 5% CO_2_. Cell proliferation assays were performed in 96-well plate. PUFAs were added with serial concentrations (20–100 μM) for 24 h. Then cells were stained with WST at 37 °C for 1 h and quantified by the absorbance at 450 nm.

### Overexpression of mfat-1 by lentiviral transfection

The description of mfat-1 cDNA has been described previously^20^. The mfat-1 cDNA was synthesized and cloned into the PLJM1 lentivirus vector (Addgene, Palo Alto, CA, USA). To produce the virus, pMD2.G and psPAX2 were co-transfected with PLJM1-mfat-1 or the vehicle plasmid into 293T cells using X-tremeGENE HP DNA transfection reagent, according to manufacturer’s instructions. To obtain a stable mfat-1 overexpressing cell line, lentivirus-containing supernatant was harvested 48 hours after transfection and used to infect RL95-2 cells.

### Quantitative analysis of gene expression levels in cells and tissues

Total RNA was prepared using TRIZOL reagent, treated with DNase I, and reverse transcribed with a Superscript kit. Real-time PCR assay was carried out on an Applied Biosystems StepOnePlus™ Systems with the following cycles: 50 °C for 2 min, one cycle; 95 °C for 30 s, one cycle; and 95 °C for 15 s, 58 °C for 30 s and 72 °C for 30 s, 40 cycles.

### Western blotting

Cells were treated with various PUFA or PGE_2_ agents at 37 °C for various times, as indicated in the experiments. The cells were collected and subjected to Western blotting analysis. The immunoreactivity was detected by using a standard enhanced-chemiluminescent reaction and analyzed using Image Lab 4.0 analysis software from Bio-Rad.

### Immunohistochemical staining

The tissue specimens were incubated with antibodies against phospho-*Akt*, Ki-67, Cyclin D1, and COX-2. The specific signals were detected using EnVision polymer technology, and visualization was performed with 3, 3N-diaminobenzidine tetrahydrochloride (DAB). The sections were photographed by Nikon microscopy and image analyses system. The expression levels of proteins were quantitated using Image Pro Plus 6.0 software (Media Cybernetics Inc., New York, USA).

### Analysis of cell cycle

After RL95-2 cells were incubated in serum-free medium overnight, cells were collected and rinsed with PBS twice. For cell cycle analysis, the cells were fixed in 70% pre-cold ethanol at 4 °C overnight and stained by PI. The DNA content was analyzed using a BD FACStar flow cytometer.

### Gas chromatography analysis of fatty acid compositions

Lipids extraction from cells and tissues were performed according to a previous report^41^. The gas chromatography was done on an Agilent 7890A. Identification of components was done by comparison of retention times with those of PUFA analytical standard.

### Systematic metabolomic analysis of eicosanoids

The RL95-2 cells were seeded in 15 cm dish 24 hours before adding various PUFA. After digesting with 0.25% trypsin, cells were washed twice by PBS and counted. 2 × 10^7^ cells were needed for eicosanoid analysis in each sample. Eicosanoid extraction was performed according to a previous report^54^. Chromatographic separation involved an ACQUITY UPLC BEH C18 column consisting of ethylene-bridged hybrid particles. The metabolites were quantified by use of a 5500 QTRAP hybrid triple quadrupole linear ion trap mass spectrometer equipped with a Turbo Ion Spray electrospray ionization (ESI) source.

### Statistical analysis

Data are presented as mean ± SD of at least three independent experiments. Differences between groups were analyzed using Student’s *t* test or one-way ANOVA, and a level of *P *< 0.05 was considered statistically significant.

## Additional Information

**How to cite this article**: Pan, J. *et al.* Elevation of ω-3 Polyunsaturated Fatty Acids Attenuates PTEN-deficiency Induced Endometrial Cancer Development through Regulation of COX-2 and PGE_2_ Production. *Sci. Rep.* **5**, 14958; doi: 10.1038/srep14958 (2015).

## Figures and Tables

**Table 1:** Analysis of ω-6 and ω-3 PUFA composition.

PUFA Species, %	aPTEN-deficiency induced endometrial cancer model				bRL95-2 cell		cxenograft mice tumors		
	PTEN ^+/+^	mfat-1	PTEN ^+/−^	mfat-1;PTEN^+/−^	RL95-2-GFP	RL95-2-mfat-1	Con	FO diet	mfat-1
ALA(C18:3 ω-3)	0.33 ± 0.05	0.46 ± 0.12	0.32 ± 0.04	0.46 ± 0.11*	0.16 ± 0.00	0.42 ± 0.01 **	0.18 ± 0.04	0.17 ± 0.06	0.47 ± 0.18
EPA(C20:5, ω-3)	0.78 ± 0.06	1.99 ± 0.31**	0.94 ± 0.38	1.85 ± 0.45**	0.25 ± 0.09	2.99 ± 0.18 **	0.02 ± 0.04	1.54 ± 0.28 **	0.48 ± 0.14 **
DPA(C22:5, ω-3)	0.71 ± 0.72	1.24 ± 0.14**	0.71 ± 0.02	1.21 ± 0.14**	2.68 ± 0.08	5.32 ± 0.05 **	0.61 ± 0.11	2.14 ± 0.48 **	1.56 ± 0.39 *
DHA(C22:6, ω-3)	5.78 ± 0.18	6.33 ± 0.42^*^	5.80 ± 0.17	6.43 ± 0.45**	1.21 ± 0.05	1.59 ± 0.01 **	1.45 ± 0.03	3.4 ± 0.67 **	2.11 ± 0.38 *
ω-3, total	7.61 ± 0.19	10.03 ± 0.53**	7.77 ± 0.43	9.96 ± 0.52**	4.29 ± 0.12	10.32 ± 0.13 **	2.26 ± 0.1	7.25 ± 1.26 **	4.62 ± 1.08 ^*^
LA(C18:2, ω-6)	17.21 ± 1.23	15.91 ± 0.55	16.93 ± 1.29	15.96 ± 0.51	1.25 ± 0.01	0.80 ± 0.07 **	15.41 ± 0.67	11.49 ± 2.63	7.16 ± 0.52 **
AA(C20:4, ω-6)	9.72 ± 0.58	7.55 ± 0.89*	9.62 ± 0.56	7.98 ± 1.32*	7.99 ± 0.12	2.37 ± 0.02 **	3.31 ± 0.78	2.14 ± 0.43 **	2.87 ± 0.39
ω-6, total	27.71 ± 1.69	24.17 ± 0.87**	27.33 ± 1.78	24.65 ± 1.41*	9.24 ± 0.11	3.17 ± 0.09 **	18.72 ± 0.51	13.63 ± 2.46 *	10.03 ± 0.14 **
ω-6/ω-3 ratio	3.64 ± 0.18	2.42 ± 0.19**	3.53 ± 0.32	2.49 ± 0.25**	2.15 ± 0.03	0.31 ± 0.01 **	8.31 ± 0.14	1.93 ± 0.52 **	2.26 ± 0.59 **

Abbreviations: ALA, α-lipoic acid; DPA, docosapentaenoic acid; LA, linoleic acid. The composition of ω-6 and ω-3 PUFAs in samples was analyzed using standard protocols (see Materials and Methods). Each species is expressed as a percentage of all fatty acid peaks, ie, the distribution areas of different ω-3 or ω-6 PUFAs peaks divided by the total peak areas of all detectable saturated and unsaturated free fatty acids (from the same sample) resolved from the gas chromatography column.

^a^the composition of ω-6 and ω-3 PUFAs in the mice blood. All data presented are means ± SD. n = 6. ^*^P < 0.05 and ^**^P < 0.01 when the mfat-1 group compared with PTEN ^+/+^ group or the mfat-1;PTEN^+/−^ group compared with PTEN ^+/−^ group.

^b^the compositions of ω-6 or ω-3 PUFAs in endometrial cancer cells.

^c^the compositions of ω-6 or ω-3 PUFAs in endometrial xenograft mice tumors. All data presented are means ± SD. n = 6. ^*^P < 0.05 and ^**^P < 0.01 compared with respective controls.

**Table 2:** Flow cytometry analysis of cell cycle in RL95-2 cells.

	G1/G0 (%)	S (%)	G2/M (%)
RL95-2-GFP	41.95 ± 1.67	42.41 ± 0.23	15.65 ± 1.88
RL95-2-mfat1	54.38 ± 1.54*	38.33 ± 0.87*	7.29 ± 1.91*

RL95-2 cells plated in 6-well plates were collected for cell cycle analysis. Data are means ± SD. n = 4.

^*^P < 0.05 compared with RL95-2-GFP.

**Table 3:** RL95-2 cell AA and DHA metabolite concentration (ng/10^5^ cell) determined by LC-MS/MS.

Oxylipin	RL95-2-GFP	RL95-2-GFP	RL95-2-GFP	RL95-2-mfat-1
		with AA	with DHA	
AA derived Epoxygenase-dependent metabolism				
14,15-EET	1.730 ± 0.556	4.131 ± 0.829**	1.237 ± 0.492	0.469 ± 0.062*
11,12-EET	1.765 ± 0.453	4.848 ± 1.394*	1.069 ± 0.488	0.371 ± 0.042^**^
8,9-EET	1.222 ± 0.249	10.286 ± 1.050**	1.164 ± 0.451	0.369 ± 0.076**
5,6-EET	0.611 ± 0.214	4.234 ± 0.369**	0.607 ± 0.238	NP
AA derived sEH-dependent metabolism				
14,15-DHET	1.696 ± 0.148	14.540 ± 2.408**	2.219 ± 0.493	0.669 ± 0.087**
11,12-DHET	0.999 ± 0.174	5.295 ± 0.814**	0.958 ± 0.239	0.417 ± 0.053**
8,9-DHET	NP	16.214 ± 1.267**	1.327 ± 0.241**	NP
5,6-DHET	3.004 ± 0.871	59.982 ± 11.910**	5.941 ± 3.860	0.331 ± 0.072**
AA derived CYPω-hydrolase-dependent metabolism				
20-HETE	1.130 ± 0.210	3.517 ± 0.294**	1.887 ± 0.550	0.724 ± 0.201
19-HETE	7.222 ± 1.380	19.954 ± 2.211**	24.640 ± 2.923**	3.513 ± 0.238*
18-HETE	4.496 ± 0.082	5.706 ± 1.498	192.263 ± 15.693**	10.526 ± 0.695**
17-HETE	0.114 ± 0.197	0.350 ± 0.112	2.651 ± 0.145**	4.431 ± 0.673**
16-HETE	0.149 ± 0.259	6.461 ± 0.636**	3.949 ± 0.522**	0.927 ± 0.085**
AA derived CYP allylic-oxidase-dependent metabolism				
11-HETE	1.506 ± 0.236	30.265 ± 5.414**	2.361 ± 0.384*	0.937 ± 0.156*
9-HETE	2.657 ± 0.952	139.155 ± 34.259**	3.832 ± 1.587	0.924 ± 0.159**
AA derived LOX-dependent metabolism				
15-HETE	2.648 ± 0.618	48.142 ± 12.248**	4.539 ± 0.830*	1.064 ± 0.012*
12-HETE	3.307 ± 0.081	120.684 ± 14.725**	5.131 ± 1.099*	0.757 ± 0.029**
8-HETE	1.487 ± 0.194	69.814 ± 8.523**	2.758 ± 0.778	0.429 ± 0.065**
5-HETE	20.574 ± 3.600	1343.25 ± 319.477**	46.108 ± 23.299	3.620 ± 0.953**
15-oxo-ETE	0.121 ± 0.050	2.731 ± 0.419**	NP	NP
5-oxo-ETE	0.467 ± 0.056	44.765 ± 12.085**	1.001 ± 0.084**	NP
LTB_4_	NP	12.850 ± 2.962**	NP	NP
LXA_4_	NP	4.986 ± 1.129**	3.595 ± 1.027**	0.427 ± 0.105**
AA derived COX-dependent metabolism				
PGE_2_	2.289 ± 0.248	3.187 ± 0.401*	0.629 ± 0.225**	0.704± 0.296**
TXB_2_	NP	NP	NP	NP
PGD_2_	NP	8.872 ± 1.451**	NP	NP
PGB_2_	NP	NP	NP	NP
PGF_2a_	NP	NP	NP	NP
PGJ_2_	NP	3.772 ± 0.679**	NP	NP
15-deoxy-PGJ_2_	NP	3.92 ± 0.976**	NP	NP
6-keto-PGF_1a_	0.051 ± 0.006	0.049 ± 0.011	0.064 ± 0.003	0.04 ± 0.001*
DHA derived LOX-dependent metabolism				
10-HDoHE	1.405 ± 0.209	2.053± 0.307*	78.16 ± 9.619**	1.073 ± 0.221
10S,17S-DiHDoHE	0.416 ± 0.13	0.523 ± 0.001	3.634 ± 1.731*	0.201 ± 0.088*
11-HDoHE	1.855 ± 0.514	3.265 ± 0.391*	132.919 ± 20.536**	1.158± 0.125
13-HDoHE	0.532 ± 0.094	0.884 ± 0.151*	27.265 ± 3.73**	0.928 ± 0.138*
14-HDoHE	0.898 ± 0.201	1.028 ± 0.207	23.025 ± 1.18**	0.486 ± 0.093*
17-HDoHE	2.261 ± 0.593	3.508 ± 1.336*	71.208 ± 15.83**	2.525 ± 0.587
20-HDoHE	2.936 ± 0.514	4.436 ± 0.941	254.062 ± 30.509*	0.393 ± 0.157*
4-HDoHE	5.963 ± 0.862	11.625 ± 4.785	706.892 ± 72.872**	6.357 ± 0.627
7-HDoHE	2.135 ± 0.45	3.722 ± 0.728*	208.913 ± 29.945**	1.069 ± 0.106*
8-HDoHE	4.184 ± 0.062	5.982 ± 0.623**	498.012 ± 57.392**	4.129 ± 0.191
Maresin	1.56 ± 0.527	1.386 ± 0.33	5.296 ± 1.703*	0.633 ± 0.127*
RvD_1_	NP	NP	NP	NP
RvD_2_	NP	NP	NP	NP
DHA derived CYP-dependent metabolism				
16,17-EDP	0.587 ± 0.102	0.705± 0.175	35.01 ± 5.652**	0.909 ± 0.191
19,20-EDP	3.076 ± 0.401	4.224 ± 0.524*	355.132 ± 65.569**	5.142 ± 0.166**

*P < 0.05, ^**^P < 0.01 compared with RL95-2-GFP; Data are mean ± SD. n = 6. NP: No peak; CYP: cytochrome P450 enzymes; COX: cyclooxygenase, LOX: lipoxygenase, PG: prostaglandin.

## References

[b1] Siegel R., Ma J., Zou Z. & Jemal A. Cancer statistics, 2014. CA Cancer J Clin 64, 9–29 (2014). 24399786 10.3322/caac.21208

[b2] Wright J. D., Barrena Medel N. I., Sehouli J., Fujiwara K. & Herzog T. J. Contemporary management of endometrial cancer. Lancet 379, 1352–1360 (2012). 22444602 10.1016/S0140-6736(12)60442-5

[b3] Dinkelspiel H. E., Wright J. D., Lewin S. N. & Herzog T. J. Contemporary clinical management of endometrial cancer. Obstet Gynecol Int 2013, 583891 (2013). 23864861 10.1155/2013/583891 PMC3707260

[b4] Tashiro H. *et al.* Mutations in PTEN are frequent in endometrial carcinoma but rare in other common gynecological malignancies. Cancer Res 57, 3935–3940 (1997). 9307275

[b5] Mutter G. L. *et al.* Altered PTEN expression as a diagnostic marker for the earliest endometrial precancers. J Natl Cancer Inst 92, 924–930 (2000). 10841828 10.1093/jnci/92.11.924

[b6] Hsu C. P. *et al.* Clinical significance of tumor suppressor PTEN in colorectal carcinoma. Eur J Surg Oncol 37, 140–147 (2011). 21194879 10.1016/j.ejso.2010.12.003

[b7] Di Cristofano A. & Pandolfi P. P. The multiple roles of PTEN in tumor suppression. Cell 100, 387–390 (2000). 10693755 10.1016/s0092-8674(00)80674-1

[b8] Chen M. L. *et al.* The deficiency of Akt1 is sufficient to suppress tumor development in Pten^+/−^ mice. Genes Dev 20, 1569–1574 (2006). 10.1101/gad.1395006 PMC1482477 16778075

[b9] Stambolic V. *et al.* High incidence of breast and endometrial neoplasia resembling human Cowden syndrome in pten^+/−^ mice. Cancer Res 60, 3605–3611 (2000). 10910075

[b10] Simopoulos A. P. The importance of the omega-6/omega-3 fatty acid ratio in cardiovascular disease and other chronic diseases. Exp Biol Med (Maywood) 233, 674–688 (2008). 18408140 10.3181/0711-MR-311

[b11] Berquin I. M., Edwards I. J. & Chen Y. Q. Multi-targeted therapy of cancer by omega-3 fatty acids. Cancer Lett 269, 363–377 (2008). 18479809 10.1016/j.canlet.2008.03.044 PMC2572135

[b12] Hajjaji N. & Bougnoux P. Selective sensitization of tumors to chemotherapy by marine-derived lipids: a review. Cancer Treat Rev 39, 473–488 (2013). 22850619 10.1016/j.ctrv.2012.07.001

[b13] de Lorgeril M. & Salen P. New insights into the health effects of dietary saturated and omega-6 and omega-3 polyunsaturated fatty acids. BMC Med 10, 50 (2012). 22613931 10.1186/1741-7015-10-50 PMC3394202

[b14] Larsson S. C., Kumlin M., Ingelman-Sundberg M. & Wolk A. Dietary long-chain n-3 fatty acids for the prevention of cancer: a review of potential mechanisms. Am J Clin Nutr 79, 935–945 (2004). 15159222 10.1093/ajcn/79.6.935

[b15] Azrad M., Turgeon C. & Demark-Wahnefried W. Current evidence linking polyunsaturated Fatty acids with cancer risk and progression. Front Oncol 3, 224 (2013). 24027672 10.3389/fonc.2013.00224 PMC3761560

[b16] Arem H. *et al.* Omega-3 and omega-6 fatty acid intakes and endometrial cancer risk in a population-based case-control study. Eur J Nutr 52, 1251–1260 (2013). 22915050 10.1007/s00394-012-0436-z PMC3548981

[b17] Terry P., Wolk A., Vainio H. & Weiderpass E. Fatty fish consumption lowers the risk of endometrial cancer: a nationwide case-control study in Sweden. Cancer Epidemiol Biomarkers Prev 11, 143–145 (2002). 11815413

[b18] Zheng H. *et al.* Inhibition of endometrial cancer by n-3 polyunsaturated fatty acids in preclinical models. Cancer Prev Res 7, 824–834 (2014). 10.1158/1940-6207.CAPR-13-0378-T 24866178

[b19] Abel S., Riedel S. & Gelderblom W. C. Dietary PUFA and cancer. Proc Nutr Soc 73, 361–367 (2014). 24850051 10.1017/S0029665114000585

[b20] Wei D. *et al.* Cellular production of n-3 PUFAs and reduction of n-6-to-n-3 ratios in the pancreatic beta-cells and islets enhance insulin secretion and confer protection against cytokine-induced cell death. Diabetes 59, 471–478 (2010). 19933995 10.2337/db09-0284 PMC2809969

[b21] Suzuki A. *et al.* T cell-specific loss of Pten leads to defects in central and peripheral tolerance. Immunity 14, 523–534 (2001). 11371355 10.1016/s1074-7613(01)00134-0

[b22] Yuan H. *et al.* 15-Lipoxygenases and its metabolites 15(S)-HETE and 13(S)-HODE in the development of non-small cell lung cancer. Thorax 65, 321–326 (2010). 20388757 10.1136/thx.2009.122747

[b23] Wen Z. H. *et al.* Critical role of arachidonic acid-activated mTOR signaling in breast carcinogenesis and angiogenesis. Oncogene 32, 160–170 (2013). 22349822 10.1038/onc.2012.47

[b24] Skrypnyk N. *et al.* PPARalpha activation can help prevent and treat non-small cell lung cancer. Cancer Res 74, 621–631 (2014). 24302581 10.1158/0008-5472.CAN-13-1928 PMC3902646

[b25] Li Z., Zhang Y., Kim W. J. & Daaka Y. PGE2 promotes renal carcinoma cell invasion through activated RalA. Oncogene 32, 1408–1415 (2012). 22580611 10.1038/onc.2012.161 PMC3421051

[b26] Zhang Y. *et al.* Combined therapy with COX-2 inhibitor and 20-HETE inhibitor reduces colon tumor growth and the adverse effects of ischemic stroke associated with COX-2 inhibition. Am J Physiol Regul Integr Comp Physiol 307, R693–703 (2014). 24990856 10.1152/ajpregu.00422.2013 PMC4214836

[b27] Nithipatikom K. *et al.* Inhibition of carcinoma cell motility by epoxyeicosatrienoic acid (EET) antagonists. Cancer Sci 101, 2629–2636 (2010). 20804500 10.1111/j.1349-7006.2010.01713.x PMC3398840

[b28] Oi N. *et al.* Resveratrol, a red wine polyphenol, suppresses pancreatic cancer by inhibiting leukotriene A(4)hydrolase. Cancer Res 70, 9755–9764 (2010). 20952510 10.1158/0008-5472.CAN-10-2858 PMC4872628

[b29] Kurtova A. V. *et al.* Blocking PGE2-induced tumour repopulation abrogates bladder cancer chemoresistance. Nature 517, 209–213 (2015). 25470039 10.1038/nature14034 PMC4465385

[b30] Damsky W. E. *et al.* beta-catenin signaling controls metastasis in Braf-activated Pten-deficient melanomas. Cancer Cell 20, 741–754 (2011). 22172720 10.1016/j.ccr.2011.10.030 PMC3241928

[b31] Macleod K. Tumor suppressor genes. Curr Opin Genet Dev 10, 81–93 (2000). 10679386 10.1016/s0959-437x(99)00041-6

[b32] Wang H. *et al.* DNA mismatch repair deficiency accelerates endometrial tumorigenesis in Pten heterozygous mice. Am J Pathol 160, 1481–1486 (2002). 11943731 10.1016/S0002-9440(10)62573-4 PMC1867211

[b33] Lin H. P. *et al.* Growth inhibitory effects of celecoxib in human umbilical vein endothelial cells are mediated through G1 arrest via multiple signaling mechanisms. Mol Cancer Ther 3, 1671–1680 (2004). 15634661

[b34] Basu G. D., Pathangey L. B., Tinder T. L., Gendler S. J. & Mukherjee P. Mechanisms underlying the growth inhibitory effects of the cyclo-oxygenase-2 inhibitor celecoxib in human breast cancer cells. Breast Cancer Res 7, R422–435 (2005). 15987447 10.1186/bcr1019 PMC1175053

[b35] Liang J. & Slingerland J. M. Multiple roles of the PI3K/PKB (Akt) pathway in cell cycle progression. Cell Cycle 2, 339–345 (2003). 12851486

[b36] Shimura T. Acquired radioresistance of cancer and the AKT/GSK3beta/cyclin D1 overexpression cycle. J Radiat Res 52, 539–444 (2011). 21881296 10.1269/jrr.11098

[b37] Caygill C. P., Charlett A. & Hill M. J. Fat, fish, fish oil and cancer. Br J Cancer 74, 159–164 (1996). 8679451 10.1038/bjc.1996.332 PMC2074598

[b38] Gago-Dominguez M., Yuan J. M., Sun C. L., Lee H. P. & Yu M. C. Opposing effects of dietary n-3 and n-6 fatty acids on mammary carcinogenesis: The Singapore Chinese Health Study. Br J Cancer 89, 1686–1692 (2003). 14583770 10.1038/sj.bjc.6601340 PMC2394424

[b39] Chavarro J. E. *et al.* A prospective study of polyunsaturated fatty acid levels in blood and prostate cancer risk. Cancer Epidemiol Biomarkers Prev 16, 1364–1370 (2007). 17585059 10.1158/1055-9965.EPI-06-1033

[b40] Jia Q. *et al.* Reduced colitis-associated colon cancer in Fat-1 (n-3 fatty acid desaturase) transgenic mice. Cancer Res 68, 3985–3991 (2008). 18483285 10.1158/0008-5472.CAN-07-6251 PMC2648804

[b41] Lu Y. *et al.* Expression of the fat-1 gene diminishes prostate cancer growth *in vivo* through enhancing apoptosis and inhibiting GSK-3 beta phosphorylation. Mol Cancer Ther 7, 3203–3211 (2008). 18852124 10.1158/1535-7163.MCT-08-0494

[b42] Weylandt K. H. *et al.* Suppressed liver tumorigenesis in fat-1 mice with elevated omega-3 fatty acids is associated with increased omega-3 derived lipid mediators and reduced TNF-alpha. Carcinogenesis 32, 897–903 (2011). 21421544 10.1093/carcin/bgr049 PMC3106436

[b43] Chen Z. *et al.* mTORC1/2 targeted by n-3 polyunsaturated fatty acids in the prevention of mammary tumorigenesis and tumor progression. Oncogene 33, 4548–4557 (2014). 24096482 10.1038/onc.2013.402

[b44] de Carvalho D. D. *et al.* Nox1 downstream of 12-lipoxygenase controls cell proliferation but not cell spreading of colon cancer cells. Int J Cancer 122, 1757–1764 (2008). 18076063 10.1002/ijc.23300

[b45] Grant G. E. *et al.* Enhanced formation of 5-oxo-6,8,11,14-eicosatetraenoic acid by cancer cells in response to oxidative stress, docosahexaenoic acid and neutrophil-derived 5-hydroxy-6,8,11,14-eicosatetraenoic acid. Carcinogenesis 32, 822–828 (2011). 21393477 10.1093/carcin/bgr044 PMC3146358

[b46] Bai X. *et al.* Prostaglandin E2 stimulates beta1-integrin expression in hepatocellular carcinoma through the EP1 receptor/PKC/NF-kappaB pathway. Sci Rep 4, 6538 (2014). 25289898 10.1038/srep06538 PMC5377465

[b47] Wu T. Cyclooxygenase-2 in hepatocellular carcinoma. Cancer Treat Rev 32, 28–44 (2006). 16337744 10.1016/j.ctrv.2005.10.004

[b48] Wendum D., Masliah J., Trugnan G. & Flejou J. F. Cyclooxygenase-2 and its role in colorectal cancer development. Virchows Arch 445, 327–333 (2004). 15340847 10.1007/s00428-004-1105-2

[b49] Vo B. T. *et al.* TGF-beta effects on prostate cancer cell migration and invasion are mediated by PGE2 through activation of PI3K/AKT/mTOR pathway. Endocrinology 154, 1768–1779 (2013). 23515290 10.1210/en.2012-2074 PMC3628025

[b50] Ohno S., Ohno Y., Suzuki N., Soma G. & Inoue M. Cyclooxygenase-2 expression correlates with apoptosis and angiogenesis in endometrial cancer tissue. Anticancer Res 27, 3765–3770 (2007). 17970040

[b51] Hasegawa K. *et al.* The effects of the selective cyclooxygenase-2 inhibitor on endometrial cytological findings in uterine endometrial cancer patients. Acta Cytol 56, 394–400 (2012). 22846758 10.1159/000338485

[b52] Hasegawa K. *et al.* Expression of cyclooxygenase-2 in uterine endometrial cancer and anti-tumor effects of a selective COX-2 inhibitor. Int J Oncol 26, 1419–1428 (2005). 15809736

[b53] Jagarlamudi K. *et al.* Oocyte-specific deletion of Pten in mice reveals a stage-specific function of PTEN/PI3K signaling in oocytes in controlling follicular activation. PLoS One 4, e6186 (2009). 19587782 10.1371/journal.pone.0006186 PMC2702689

[b54] Li L. *et al.* Opposite effects of gene deficiency and pharmacological inhibition of soluble epoxide hydrolase on cardiac fibrosis. PLoS One 9, e94092 (2014). 24718617 10.1371/journal.pone.0094092 PMC3981766

